# Co-occurrence of Attention-Deficit/Hyperactivity Disorder and Septo-Optic Dysplasia: A Neurodevelopmental Case Report

**DOI:** 10.7759/cureus.60441

**Published:** 2024-05-16

**Authors:** Aliya Centner, Safia Centner, Jamie Ikeda, Leslie A Gavin, Shirin Hasan

**Affiliations:** 1 Medicine, University of Central Florida College of Medicine, Orlando, USA; 2 Ophthalmology, Nemours Children's Hospital, Orlando, USA; 3 Child Psychology, Nemours Children's Hospital, Orlando, USA; 4 Child and Adolescent Psychiatry & Pediatrics, Nemours Children's Hospital, Orlando, USA

**Keywords:** congenital birth defect, neurodevelopmental disorders, neurodevelopmental assessment, attention-deficit/hyperactivity disorder (adhd), septo-optic dysplasia

## Abstract

Septo-optic dysplasia (SOD) is a rare congenital disorder characterized by optic nerve hypoplasia, brain midline structure anomalies, and hypothalamic-pituitary axis hypoplasia. This case report aims to highlight the association between SOD and neurodevelopmental disorders, focusing on attention-deficit/hyperactivity disorder (ADHD) in addition to the well-established link with autism spectrum disorder (ASD). A six-year-old male diagnosed with SOD presented with behavioral concerns, including attention and impulse control issues. A comprehensive psychological evaluation confirmed the diagnosis of ADHD and ruled out ASD. Ophthalmological assessments were integral to understanding the patient's condition. This case underscores the importance of recognizing neurodevelopmental disorders in individuals with SOD, with a particular focus on the less common association with ADHD. The co-occurrence of these conditions underscores the complexity of neurodevelopmental disorders and the need for comprehensive evaluation and management. Collaboration between ophthalmologists and mental health specialists is crucial for addressing the diverse needs of these patients. Early identification and intervention for ADHD are essential for optimal developmental outcomes. This case underscores the necessity for further research to elucidate the relationship between SOD and ADHD, emphasizing the importance of holistic patient care and interdisciplinary collaboration in managing individuals with SOD spectrum conditions.

## Introduction

Septo-optic dysplasia (SOD), also known as de Morsier’s syndrome, is a rare congenital disorder that is characterized by ophthalmic, neurologic, and endocrine disturbances. The classic triad consists of optic nerve hypoplasia (ONH) or dysplasia, agenesis of brain midline structures (septum pellucidum, corpus callosum, and others), and hypoplasia of the hypothalamic-pituitary axis. Patients should have at least two of the three features in order to meet the criteria for a diagnosis. Ophthalmic disturbances include decreased visual acuity, amblyopia, refractive error, astigmatism, strabismus, or nystagmus. Neurological disturbances include cerebral palsy, hemiparesis, seizures, and developmental delays. Patients may also have hypopituitarism, including panhypothyroidism, diabetes insipidus, adrenal insufficiency, hyperprolactinemia, micropenis, or pubertal delay. [1]

The SOD-plus syndrome refers to a diagnosis of SOD in addition to cortical malformations including schizencephaly, polymicrogyria, and gray matter heterotopias [2]. The SOD spectrum refers to individuals with a larger range of congenital anomalies, including a cleft palate, or with more endocrine deficiencies and less visual impairment [1]. 

The etiology of SOD is unknown but may be associated with genetic predisposition or prenatal environmental factors such as toxins and pollution [1]. Most cases of SOD are considered sporadic. However, some cases have been found to be associated with mutations in the HESX1, SOX2, SOX3, and OTX2 genes, which encode developmental transcription factors that are involved in forebrain and pituitary development [3]. The HESX1 gene has been found to be associated with endocrine deficiencies; OTX2 and SOX2 genes are involved in the embryonic development of the optic nerves [4]. The incidence of SOD is estimated to be one in 10,000 [1], and a study found the prevalence of SOD in Europe to be 1.9 to 2.5 per 100,000 births [5].

In general, non-progressive visual impairment is the most common presentation. However, individuals with SOD may also exhibit minimally impaired vision while being more affected by other midline defects or endocrine deficiencies, resulting in a broad range of presentations [1]. The majority of children diagnosed with SOD are born at full term, showing appropriate size and a good appearance, pulse, grimace, activity, and respiration (APGAR) score at birth. Common pathologic neonatal signs in SOD include hypoglycemia and hyperbilirubinemia during the first week of life [1].

Studies on SOD spectrum conditions often highlight neurodevelopmental impairments, with a focus on developmental delays that can vary from specific deficits, such as motor impairment, to more widespread delays [6]. Developmental and psychomotor delays have been observed in approximately 60% of individuals with SOD [1]. These delays often contribute to difficulties in social communication, accompanied by stereotypical behaviors such as motor stereotypies and echolalia, along with deficits in peer interactions. Reports have found that the prevalence of neurodevelopmental disorders in individuals with SOD is likely high [6]. 

Autism spectrum disorder (ASD) is a neurodevelopmental condition characterized by challenges in social communication and the presence of restrictive and repetitive behaviors [6]. Autism spectrum disorder can be present in around 30% to 36% of individuals with SOD [1]. Autism spectrum disorder can be diagnosed by trained clinicians via a thorough review of developmental and behavioral history and an evaluation of communication skills and social interaction [7]. The Autism Diagnostic Observation Schedule, Second Edition (ADOS-2) is a test administered to individuals as a part of a comprehensive evaluation [7]. In the test, the individual is presented with activities and tasks, and their behavior is rated in terms of communication, social interaction, and restricted and repetitive behaviors [7]. The ADOS-2 is currently considered a “gold standard” for ASD diagnosis [7].

Attention-deficit/hyperactivity disorder (ADHD) is a neurodevelopmental disorder characterized by persistent patterns of inattention, hyperactivity, and impulsivity that can interfere with daily functioning or development [8]. Individuals may struggle with sustaining attention, following through on tasks, and organizing activities. They may appear forgetful and easily distracted. They may have excessive fidgeting, restlessness, impulsivity, and difficulty waiting their turn. The Behavior Rating Inventory of Executive Function, Second Edition (BRIEF-2) is a questionnaire measure that assesses executive functioning, including all aspects of cognitive, behavioral, and emotional control. This includes the ability to inhibit behavior and self-monitor, the ability to control emotional outbursts, and the ability to the ability to manage new experiences [9]. The Achenbach System of Empirical Based Assessment (ASEBA) is a set of questionnaire measures assessing behavioral, emotional, social, and thought problems in adaptive functioning that can help assess ADHD in children and adolescents [10]. A review of the literature has found that while ASD has commonly been associated with SOD, there are fewer reports of ADHD and its association with SOD.

This article will be presented as a poster at the 2024 Women in Ophthalmology Summer Symposium on August 22-25, 2024, in Carlsbad, California.

## Case presentation

A male patient with a medical history of SOD was evaluated by psychiatry at age six for behavioral concerns. Behavioral concerns included issues with attention, focus, concentration, and the inability to stay on task. He was impulsive, intrusive, oppositional, defiant, argumentative, and easily frustrated. The patient was diagnosed with ADHD and started on methylphenidate hydrochloride extended-release. His grandmother noted that it helped some in school but caused agitation and aggression, especially in the evenings. Because the patient was receiving his ophthalmology care with us and her concerns about his behaviors arose, the guardian requested the transfer of care to our psychiatry clinic.

The patient had been diagnosed with ONH around six to eight months of age. The patient was referred for an ophthalmology evaluation at that time for wandering eye movements and poor focus. A brain MRI at 14 months of age revealed marked hypoplasia of the optic nerves bilaterally and the absence of septum pellucidum. The patient was diagnosed with SOD. He was referred to an endocrine clinic and seen at 20 months. There was no sign of pituitary dysfunction, although labs did show slight insulin-like growth factor 1 (IGF-1) elevation. However, over the next couple of years, the patient’s growth continued to be on track with a normal growth velocity. He was monitored by ophthalmology, and an initial evaluation done at two years of age in our ophthalmology clinic verified his diagnosis of SOD. His examination is shown below in Tables 1-3.

**Table 1:** Preverbal visual acuity Visual acuity of the right and left eye as demonstrated by near without correction and intraocular pressure. Confrontational visual fields are included but were not able to be assessed due to the patient's age.

	Right eye (OD)	Left eye (OS)
Near without correction	Fixation central, unsteady, and maintained	Fixation central, unsteady, not maintained
Intraocular pressure (method: finger tensions)	Normal to palpation	Normal to palpation
Confrontational visual fields	Not able to assess due to patient's age	Not able to assess due to patient's age

**Table 2:** Muscle balance This table illustrates the findings related to distance and near vision, orthotropia, fixation preference, ocular motility, stereoacuity, head postures, and nystagmus.

Distance method: Hirschberg, limited cover test	Orthotropia
Near method: Hirscriberg limited cover test	Orthotropia, possible Intermittent left esotropia (poor cooperation)
Fixation preference	Right eye (OD)
Ocular motility	Full versions
Stereoacuity (Titmus)	Unable to test
Head postures	Face turn to the right with a slight chin down
Nystagmus	Present bilaterally, moderate amplitude, horizontal jerk nystagmus with latent component

**Table 3:** Fundus examination Fundoscopic examination was performed with an indirect ophthalmoscope with a 2.2D lens.

	Right eye (OD)	Left eye (OS)
Vitreous	Clear	Clear
Optic nerve	Moderate hypoplasia, estimated 80% nerve tissue present in the scleral window	Severe hypoplasia, estimated 30% nerve tissue present in the scleral window
Cup to disc (C/D) ratio	0.6 (difficult assessment due to dysplasia)	Cannot assess due to dysplasia
Macula	Normal, good foveal reflex	Normal, good foveal reflex
Vessels	Normal caliber, no tortuosity	Normal caliber, no tortuosity
Periphery	Normal	Normal

The patient had no other major medical health issues except for mild asthma.

On reassessment with psychiatry at six years of age, the patient’s maternal grandmother, who had legal custody of the patient from the age of two due to the inability of the parents to provide care, indicated there were a lot of psychosocial stressors in the family as well as behavioral concerns. As noted above, the behavioral concerns included issues with attention, concentration, focus, impulsivity, and aggression with current medications that cause side effects. The family history was positive for depression and substance abuse. The prenatal course had no substance abuse but had been complicated by gonorrhea in the second trimester, which was treated with antibiotics. The patient was born at 38 weeks gestation with a birth weight of five pounds, 13 ounces. No problems were noted at birth. There were no developmental concerns. 

The patient's ADHD diagnosis was confirmed with a history, an exam, and the ASEBA. The patient’s medication was changed from methylphenidate hydrochloride extended-release to lisdexamfetamine, with significant improvement in behaviors and no side effects. The patient also continued behavioral therapy.

Because of the known association of ASD with SOD and some concerns from the grandmother about poor social skills and sensory issues, a psychological evaluation for ASD was recommended. However, because the patient responded well to therapy and medication, the grandmother decided not to pursue the psychological evaluation initially.

The patient did well for several years on his current ADHD medication. However, escalating behavior issues and the patient moving from the grandmother’s care to the mother’s care resulted in psychological testing being eventually administered to clarify the diagnostic picture. This evaluation was completed when the patient was 12 years old. He had psychological testing including the Wechsler Intelligence Scale for Children® Fifth Edition (WISC®-V), the Woodcock-Johnson IV Tests of Cognitive Abilities, the Woodcock-Johnson IV Tests of Achievements, the BRIEF-2 Score, and ADOS-2.

His WISC®-V performance, shown in Table 4, placed him in the average range of intelligence. Psychology determined that this was a valid assessment of his current cognitive functioning, although he did have a high degree of scatter among his scores with a particular weakness in processing speed.

**Table 4:** Wechsler Intelligence Scale for Children® Fifth Edition A score within the range of 90-109 places the patient in the average range.

Verbal comprehension	106	Average
Visual-spatial	118	Above average
Fluid reasoning	97	Average
Working memory	110	Above average
Processing speed	77	Borderline
Full scale	102	Average

Woodcock-Johnson IV Tests of Cognitive Abilities was utilized to assess the patient’s cognitive abilities [11]. His short-term working memory was in the average range, his long-term working memory was in the low average range, his auditory processing was in the average range, and his cognitive efficiency was in the low range. Woodcock-Johnson IV Tests of Achievements was utilized to assess math, reading, and writing skills. There was no sign of a specific learning disability. 

The BRIEF-2 Score, shown in Table 5, was utilized to assess executive functioning, which includes all aspects of cognitive, behavioral, and emotional control. The following table represents the parent's view of his ability to manage his behavior, emotions, and cognitive functioning, including attention, organization, and short-term memory. The mother reported clinically significant concerns in all of the subscales, except the organization of materials scale, where the score indicated at-risk concerns. The patient’s mother perceived clinically significant concerns in his ability to inhibit, self-monitor, shift, control emotions, initiate, work memory, plan/organize, task-monitor, and organize materials.

**Table 5:** Behavior Rating Inventory of Executive Function, Second Edition (BRIEF-2) **clinically significant; *at risk T-scores at or above 70 are considered clinically significant, while t-scores above 60 should be considered at risk.

		Parent
	Description	T-score
Inhibit	Ability to inhibit behavior	84**
Self-monitor	Ability to self-monitor	78**
Behavior regulation index	Includes inhibit and self-monitor	84**
Shift	Ability to manage new experiences	79**
Emotional control	Ability to control emotional outbursts	80**
Emotional regulation index	Includes shift and emotional control	81**
Initiate	Ability to begin a task or activity and to independently generate ideas, responses, or problem-solving strategies	79**
Working memory	Difficulty remembering a list of tasks, short attention span, difficulty with multi-step directions, difficulty finishing tasks and other similar items that require holding information in one’s mind, doing them without losing track or forgetting	85**
Plan/organize	Becoming easily overwhelmed by tasks and underestimating the time needed to complete tasks	77**
Task-monitor	Doing sloppy work, loving work incomplete, and making careless errors	73**
Organization of materials	Losing and forgetting things	67*
Cognitive regulation index	Includes initiative, working memory, plan/organize, task-monitor, and organization of materials	80**
Global executive composite	Includes all	86**

The ADOS-2, shown in Table 6, was utilized to assess for a diagnosis of ASD. The patient was found to be classified as not being on the autism spectrum.

**Table 6:** Autism Diagnostic Observation Schedule, Second Edition (ADOS-2) A score of nine or above exceeds the threshold for autism spectrum disorder.

Social affect (SA) subscale	0
Restricted and repetitive behavior (RRB)	0
Total	0

This testing confirmed the diagnosis of ADHD with some depressive symptoms and ruled out ASD.

## Discussion

This case emphasizes a less common and less well-known association between SOD and ADHD, as compared to the well-established link with ASD. It is important for providers to be aware of the potential association between SOD and ADHD which is highlighted by our case. The limited prevalence and literature on this association underscore the need for further research to better understand the relationships between SOD and various neurodevelopmental disorders, particularly ADHD.

Recognizing the association between SOD and neurodevelopmental disorders is crucial for comprehensive patient care. Neurodevelopmental disorders, including ASD and ADHD, may coexist with SOD [12]. Screening for these conditions in patients with SOD is essential to address the varied challenges associated with neurodevelopmental disorders and provide appropriate interventions and support. Currently, there is a lack of regular assessment for neurodevelopmental impairments, resulting in untreated conditions [6]. Clinicians typically address these issues only when caregivers express challenges in managing their child's behavior at home and school [6]. Increasing awareness of neurodevelopmental impairments in children with SOD spectrum conditions is expected to facilitate the development of early intervention strategies and treatment plans [6]. 

The behavioral factors associated with SOD can impact cooperation during ophthalmology treatment. Recognizing these behavioral aspects is crucial for ophthalmologists to provide patient-centered care. Collaboration between ophthalmologists and other specialists, particularly mental health specialists and psychiatrists, is essential to address the diverse needs of individuals with SOD [4]. Given the potential behavioral challenges associated with SOD, ophthalmologists should be attentive and consider referrals to psychiatry when necessary. Collaborative care ensures that individuals with SOD receive comprehensive support, addressing both ophthalmological and mental health aspects.

Mental health professionals, including psychiatrists and psychologists, should be aware of the association between SOD and ASD to provide the best patient care [4]. Additionally, they should be vigilant about screening for ADHD in individuals with SOD. This dual awareness is crucial for providing holistic mental health care that addresses the unique challenges associated with both SOD and coexisting neurodevelopmental disorders. Early identification and intervention for ADHD, if present, can be particularly important for an optimal developmental outcome.

Children within the spectrum of SOD encounter neurodevelopmental challenges, necessitating intervention from appropriately trained clinicians like psychiatrists or clinical psychologists [6]. The integration of psychological assessments for ASD as well as ADHD into routine care for children with SOD facilitates the diagnosis, which is crucial for therapeutic intervention by clinicians and educational support in schools. Awareness of the association between neurodevelopmental impairments and the condition aids in early diagnosis, enabling intervention at an earlier developmental stage. This will help to enhance the prognosis for children with SOD spectrum conditions, subsequently minimizing educational and behavioral challenges for both patients and caregivers and leading to improvements in their overall quality of life [6].

## Conclusions

Recognizing the link between SOD and neurodevelopmental disorders is crucial for comprehensive patient care. Septo-optic dysplasia may coexist with neurodevelopmental conditions like ASD and ADHD, necessitating screening to address associated challenges. The case highlights a less common association with ADHD compared to ASD, underscoring the need for further research. Behavioral factors related to SOD can impact cooperation during ophthalmology treatment, emphasizing collaboration between ophthalmologists and mental health specialists. Mental health professionals should be aware of SOD's association with ASD and the need to screen for ADHD, ensuring holistic care for patients and early intervention for improved overall well-being.
